# The zebrafish orthologue of the human hepatocerebral disease gene *MPV17* plays pleiotropic roles in mitochondria

**DOI:** 10.1242/dmm.037226

**Published:** 2019-03-14

**Authors:** Laura Martorano, Margherita Peron, Claudio Laquatra, Elisa Lidron, Nicola Facchinello, Giacomo Meneghetti, Natascia Tiso, Andrea Rasola, Daniele Ghezzi, Francesco Argenton

**Affiliations:** 1 Department of Biology, University of Padova, Via Ugo Bassi, 58/B, 35131 Padova, Italy; 2 Department of Biomedical Sciences, University of Padova, Via Ugo Bassi, 58/B, 35131 Padova, Italy; 3 Unit of Medical Genetics and Neurogenetics, Fondazione IRCCS Istituto Neurologico Carlo Besta, Via Amadeo 42, 20133 Milan, Italy; 4 Department of Pathophysiology and Transplantation, University of Milan, Via Libero Temolo 4, 20126 Milan, Italy

**Keywords:** Dihydroorotate dehydrogenase, Iridophore, Mitochondrial DNA depletion syndrome, Pyrimidine, Vitamin B13

## Abstract

Mitochondrial DNA depletion syndromes (MDS) are a group of rare autosomal recessive disorders with early onset and no cure available. MDS are caused by mutations in nuclear genes involved in mitochondrial DNA (mtDNA) maintenance, and characterized by both a strong reduction in mtDNA content and severe mitochondrial defects in affected tissues. Mutations in *MPV17*, a nuclear gene encoding a mitochondrial inner membrane protein, have been associated with hepatocerebral forms of MDS. The zebrafish *mpv17* null mutant lacks the guanine-based reflective skin cells named iridophores and represents a promising model to clarify the role of Mpv17. In this study, we characterized the mitochondrial phenotype of *mpv17^−/−^* larvae and found early and severe ultrastructural alterations in liver mitochondria, as well as significant impairment of the respiratory chain, leading to activation of the mitochondrial quality control. Our results provide evidence for zebrafish Mpv17 being essential for maintaining mitochondrial structure and functionality, while its effects on mtDNA copy number seem to be subordinate. Considering that a role in nucleotide availability had already been postulated for MPV17, that embryos blocked in pyrimidine synthesis do phenocopy *mpv17^−/−^* knockouts (KOs) and that *mpv17^−/−^* KOs have impaired Dihydroorotate dehydrogenase activity, we provided *mpv17* mutants with the pyrimidine precursor orotic acid (OA). Treatment with OA, an easily available food supplement, significantly increased both iridophore number and mtDNA content in *mpv17*^−/−^ mutants, thus linking the loss of Mpv17 to pyrimidine *de novo* synthesis and opening a new simple therapeutic approach for *MPV17*-related MDS.

## Abstract (precis)

**Summary:** The zebrafish *mpv17*^−/−^ mutant shows a severe mitochondrial phenotype with ultrastructural alterations and oxidative phosphorylation impairment. The pyrimidine precursor orotic acid ameliorates *mpv17*^−/−^ phenotype and increases mitochondrial DNA content, linking the loss of Mpv17 to pyrimidine *de novo* synthesis.

## INTRODUCTION

In the past few years, the scientific scenario identified mitochondria as key players in a growing spectrum of human diseases. Beside their main functions in oxidative phosphorylation and bioenergetics, mitochondria also participate in a plethora of cell pathways, such as apoptosis, production of reactive oxygen species (ROS), heat generation and lipid metabolism (Wallace, 2005). Notably, mitochondrial functionality requires a synergistic relationship between mitochondrial and nuclear genomes (Chinnery and Hudson, 2013). In fact, mitochondrial DNA (mtDNA) encodes only 13 subunits of the respiratory chain (RC), while the remaining ∼1500 mitochondrial proteins, encoded by nuclear DNA (nDNA), are imported into the organelle (Calvo et al., 2016).

Each mitochondrion may contain hundreds of copies of mtDNA and, considering that energy demand depends on age, tissue and life style, even in physiological conditions, the amount of mtDNA differs between cell types and individuals (Suomalainen and Isohanni, 2010). What has become clear, however, is that having the correct mtDNA amount in each tissue is crucial. In fact, mtDNA depletion has been discovered to cause a continuously increasing group of severe human diseases called mtDNA depletion syndromes (MDS), first described in 1991 (Moraes et al., 1991).

MDS are a clinically heterogeneous group of autosomal-recessive mitochondrial disorders, caused by mutations in nuclear genes, such as *MPV17*, involved in mtDNA replication and maintenance. Symptoms of MDS typically appear during infancy and childhood, and are characterized by the reduction of mtDNA copy number in the affected tissues (Nogueira et al., 2014). MPV17 is an inner-membrane mitochondrial protein possibly involved in mitochondrial deoxynucleoside triphosphates (dNTP) pool homeostasis (Löllgen and Weiher, 2014; Krauss et al., 2013). Mutations in the *MPV17* gene are known to cause Navajo neurohepatopathy (NNH) (Karadimas et al., 2006) and a hepatocerebral form of MDS (Spinazzola et al., 2006). In fact, of ∼50 *MPV17* pathogenic variants reported worldwide in ∼100 patients, the c.149G>A (*p.*R50Q) missense mutation has been found with an unusually high frequency in NNH-affected individuals (El-Hattab et al., 2018). To date, the precise function of MPV17 is unclear, even though many studies have been carried out in different *in vivo* models. Among those, two spontaneous zebrafish *mpv17* mutants, *roy orbison* (*roy*) and *transparent* (*tra*), have been independently identified (Krauss et al., 2013; D'Agati et al., 2017). Notably, although the genomic lesions have not been properly sequenced so far in each mutant line, they both lead to the same 19 bp deletion in the coding sequence, originating from a defective splicing of intron 2. Both *roy* and *tra* (*mpv17*^−/−^) display pigmentation abnormalities due to the lack of cells derived from the neural crest, such as iridophores, and, at later stages of development, melanophores (Krauss et al., 2013). Notably, in vertebrates, neural crest cells migrate and give rise to a large variety of complex structures, including peripheral nervous system, part of the craniofacial skeleton and pigment cells (Kelsh et al., 1996). In particular, the zebrafish pigment pattern presents three distinct types of cells: black melanophores, yellow xantophores and reflective iridophores, which strongly interact with each other and are distributed along longitudinal stripes and silvery/yellow interstripes (Kelsh et al., 2017). Several zebrafish mutants bearing recessive mutations in the *de novo* purine synthesis genes *gart* and *paics* show defects in iridophores, the cells containing light-reflective purine platelets, which are responsible for the shiny appearance of the striped pattern (Frohnhöfer et al., 2013; Ng et al., 2009).

In this work, we validated the functional homology between human and zebrafish Mpv17 and improved the characterization of the *roy/tra* phenotype, providing evidence of mitochondrial defects, RC impairment and mtDNA depletion. Our findings indicate *roy* and *tra* as valuable models for *MPV17*-related MDS. Moreover, because the chemical inhibition of pyrimidine *de novo* synthesis causes iridophore and melanophore loss in zebrafish embryos (White et al., 2011), we investigated the function of *mpv17* by administrating dNTPs and pyrimidine precursors to *mpv17^−/−^* mutants, and found significant impairment of Dihydroorotate dehydrogenase (Dhodh) activity in mutants.

## RESULTS

### Mpv17 and its paralogue, Mpv17-like2, crosstalk in *mpv17^−/−^* larvae

**Fig. 1.**
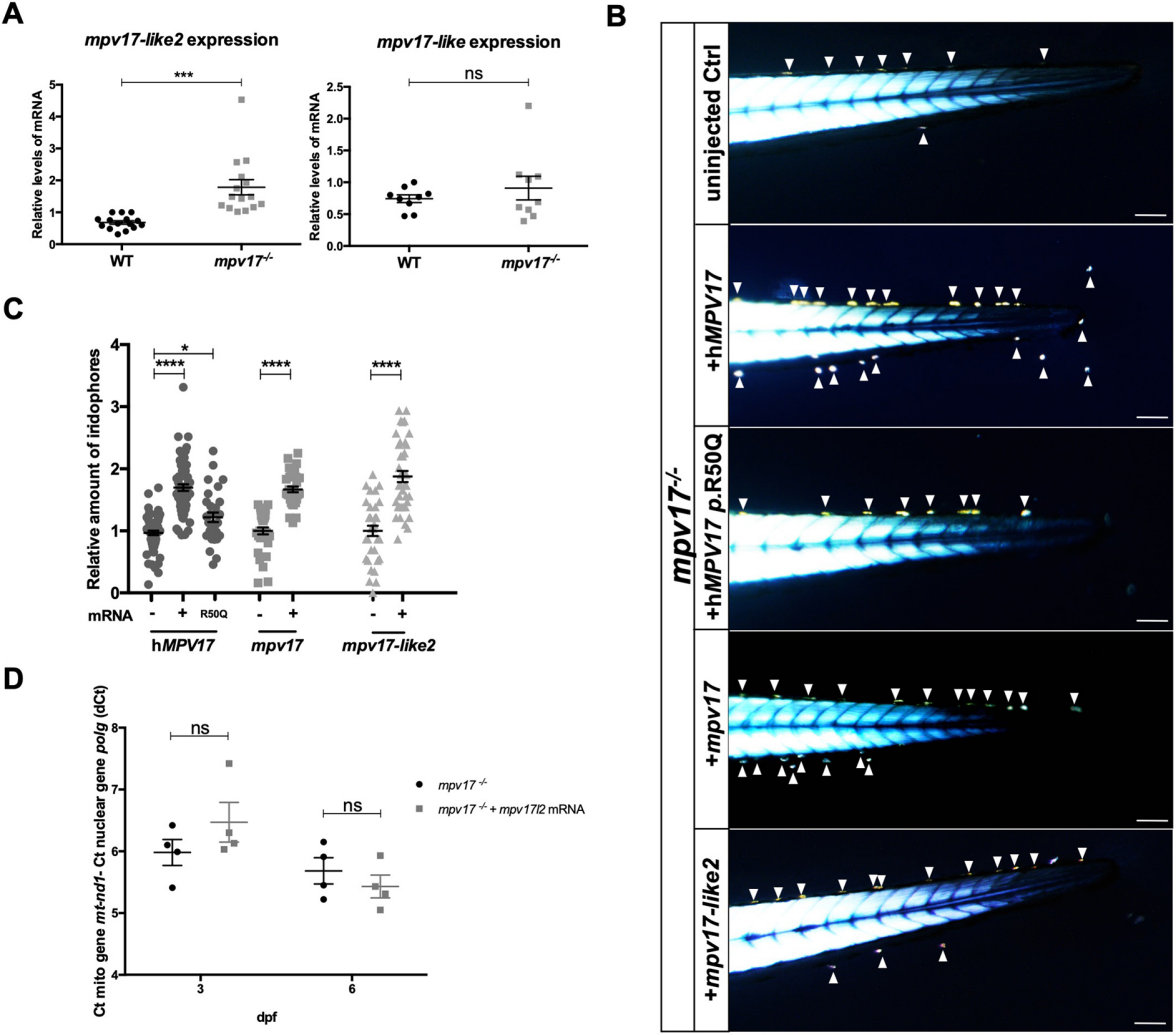
**Investigation of *mpv17* orthologue and paralogue genes in zebrafish larvae.** (A) Real-time PCR quantification of mRNA transcripts of *mpv17-like2* and *mpv17-like* at 6 dpf (*n*=9). (B) Evaluation of phenotypic rescue in the tail region at 3 dpf after the injection of human *MPV17* mRNAs, wild-type (WT) and p.R50Q mutated forms, and zebrafish *mpv17* and *mpv17-like2* mRNAs. Arrowheads point to iridophores. Scale bars: 100 µm. (C) Relative quantification of iridophore amount in controls and injected larvae (*n*=30). (D) mtDNA copy number analysis in *mpv17^−/−^* mutants transiently overexpressing *mpv17-like2* at 3 dpf and 6 dpf. Mean dCt values were calculated as Ct of *mt-nd1* (mitochondrially encoded gene) minus Ct of *polg* (nuclear gene) (*n*=4). Statistical analyses were performed using two-tailed Student's *t*-test. Statistical significance was evaluated by setting a confidence interval of 95%; data are mean±s.e.m. ****P<0.0001; ***P<0.001; *P<0.05; ns, not significant. Experiments were performed in biological replicates.

Because zebrafish *mpv17* null mutants are viable and fertile, we verified whether *mpv17* paralogue genes might play compensatory activities in *mpv17*^−/−^ mutants, thus ameliorating their phenotype. By performing reverse transcription and quantitative polymerase chain reaction (RT-qPCR) analysis, we observed a significant overexpression of *mpv17-like2* (*mpv17l2*), whereas the *mpv17-like* (*mpv17l*) expression level remained unchanged in both wild-type and homozygous knockout (KO) larvae at 6 days post-fertilization (dpf) (Fig. 1A). This result was also confirmed by *in situ* hybridization (ISH), which showed wider *mpv17l2* expression in *mpv17*^−/−^ mutant larvae at 3 dpf, compared with the liver-specific localization observed in wild-type controls (Fig. S1). Hence, we checked whether *mpv17l2* transcripts might rescue the *mpv17* null mutant phenotype, and thus whether their protein functions could overlap. To this aim, we injected one-cell-stage embryos with mRNAs encoding zebrafish *mpv17* and *mpv17l*2, and wild-type and *p.*R50Q human *MPV17* (Fig. 1B), and evaluated iridophore number at 3 dpf. Early phenotyping of *mpv17*^−/−^ zebrafish mutants can be easily performed with the aid of a microscope equipped with two polarizing filters and using the birefringence property of iridophores. Briefly, embryos are anaesthetized, flat oriented along the lateral axis and placed above the polarizer lens in a transmitted-light-dissecting microscope. Then, according to published protocols for birefringence analysis (Smith et al., 2013), the polarization plan of the analyser lens, placed above the sample, is oriented at 90° with the polarizer. With this procedure, the light from the background is completely abolished, leaving a black surround. This application of birefringence, in contrast with Krauss et al. (2013), allows the precise counting of iridophore number independently from the orientations of light and samples (Fig. S2). In particular, iridophores are readily visible at 3 dpf; they are the first metamorphic chromatophores to become observable in the dermis of the lateral trunk, and appear only later in the anterior trunk in a segmental fashion, continuing to increase in number and to accumulate to form the first ventral interstripe (Higdon et al., 2013; Frohnhöfer et al., 2013).

Interestingly, in all injected samples, except for *p*.R50Q-injected larvae, we could observe a significant and comparable increase in the number of iridophores. Notably, injection of mRNA encoding the human *p.*R50Q mutation had a significantly lower effect on the amount of iridophores (Fig. 1C). Hence, we performed an mtDNA copy number analysis on *mpv17l*2-injected embryos and found a non-significant increase in mtDNA content at 3 dpf (Fig. 1D).

Together, these data show that *mpv17* and *mpv17l2* functions may partially overlap, and demonstrate a similar function for human and zebrafish *mpv17*, confirming *roy* as a sound model for investigating *mpv17* roles.

### Loss of Mpv17 alters mitochondrial ultrastructure and respiratory complex stability

**Fig. 2.**
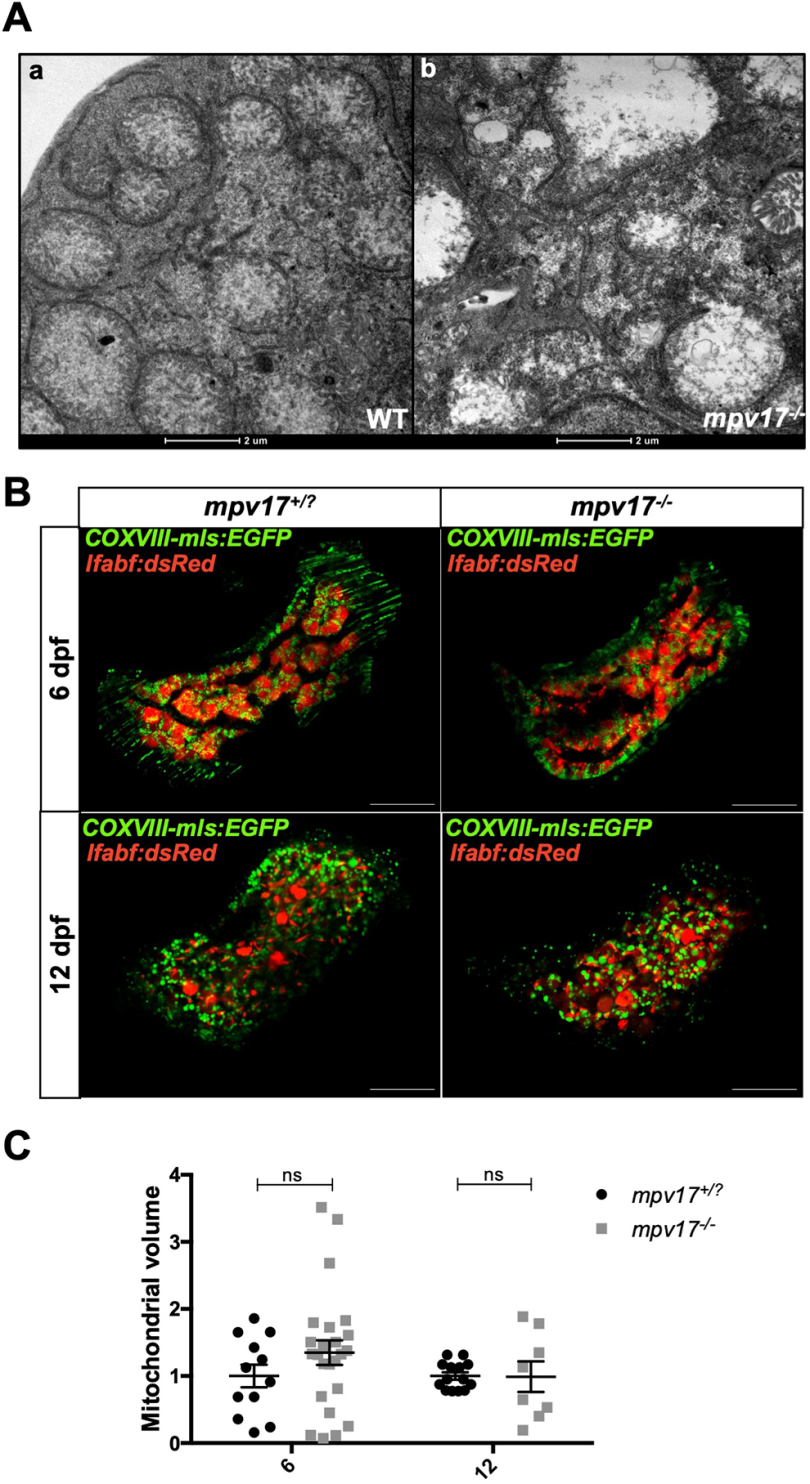
**Analysis of mitochondrial ultrastructure and volume in *mpv17^−/−^* and wild-type liver at 6** **dpf.** (A) TEM analysis of wild-type (a) and *mpv17^−/−^* liver hepatocytes (b) (*n*=3). Scale bars: 2 µM. (B) Confocal images of double *Tg(COXVIII-mls:EGFP)*; *Tg(lfabp:dsRed) mpv17^+/?^* and *mpv17^−/−^* siblings at 6 dpf (*n*=13) and 12 dpf (*n*=8). Scale bars: 50 µm. (C) Relative quantification of integrated density. Statistical analyses were performed using two-tailed Student's *t*-test. Statistical significance was evaluated by setting a confidence interval of 95%; data are mean±s.e.m. ns, not significant. Experiments were performed in biological replicates.

*MPV17*-related MDS primarily show hepatic manifestations and patients die of liver failure between 6 and 28 months of age (Kim et al., 2016). To characterize the *mpv17*^−/−^ mutant phenotype, we investigated the liver mitochondrial morphology of *mpv17*^−/−^ larvae, by transmission electron microscopy (TEM) analysis. Results showed a tissue-specific disruption of mitochondrial ultrastructure in liver hepatocytes at 6 dpf, with mitochondrial ballooning and disappearance of cristae (Fig. 2A); this phenotype was absent at 3 dpf and no difference was detected in brain and muscle tissues (data not shown). In order to assess whether an impairment of mitochondrial morphology might modify the amount of mitochondria in the liver, we crossed *mpv17^−/−^* mutants with two different transgenic lines expressing, respectively, a ubiquitous mitochondrially targeted enhanced green fluorescent protein (EGFP) and a cytosolic red fluorescent protein (dsRed) under the control of a hepatic promoter. In this way, we could analyse mitochondria only where the red fluorescence was decorating the liver. When comparing heterozygous and homozygous siblings at 6 dpf and 12 dpf (Fig. 2B), we did not observe any difference in the total volume of hepatocytes (data not shown) and liver mitochondria (Fig. 2C).

**Fig. 3.**
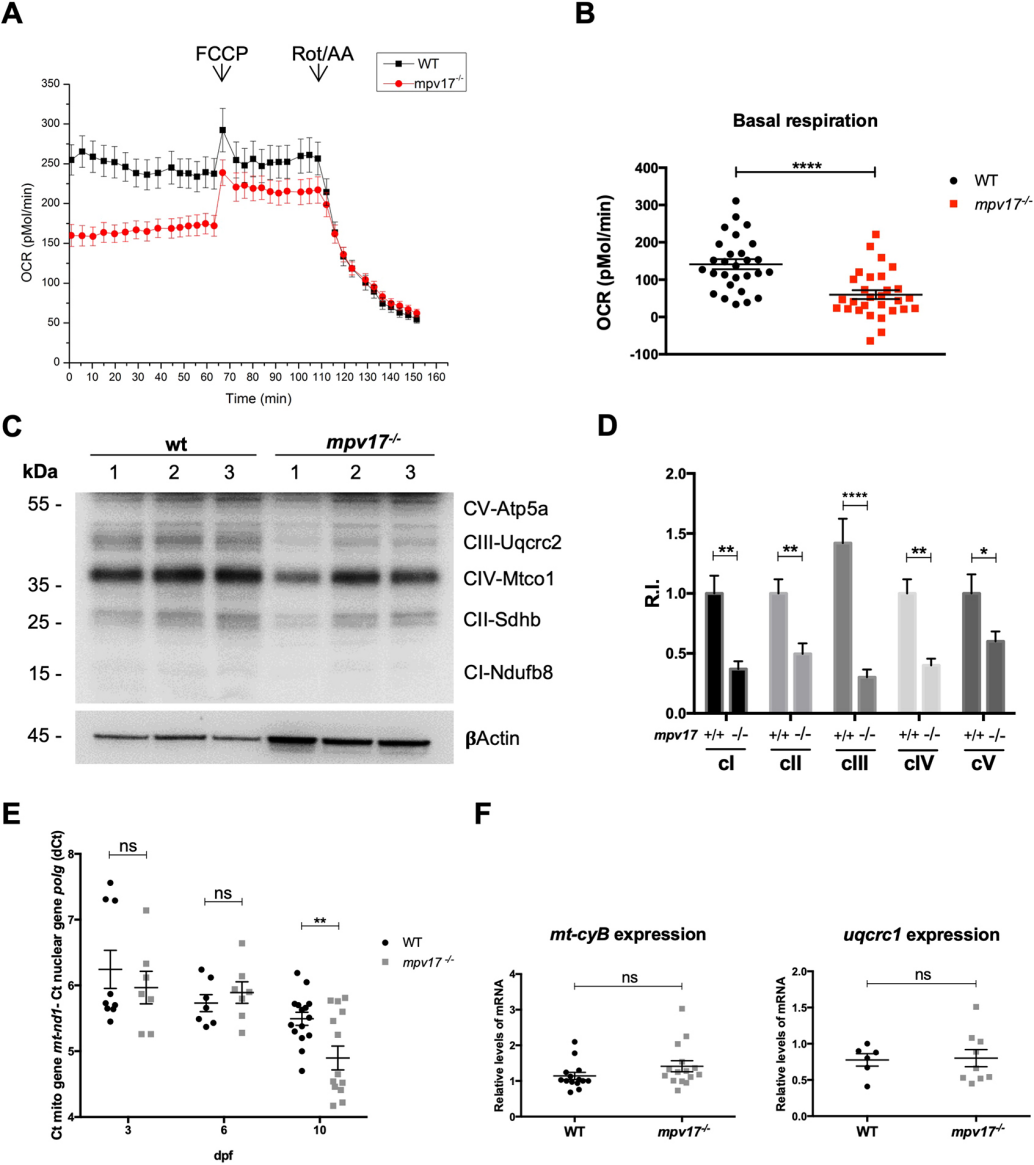
**Biochemical and genetic characterization of mitochondrial phenotype of *mpv17* KO larvae.** (A) Basal OCR was measured for ∼1 h in fish water, immediately after 4 dpf larvae were exposed to 0.5 μM FCCP and, later, to a combination of 2 μM rotenone (Rot) and 5 μM antimycin (AA). Four independent experiments were performed (*n*=40). (B) Quantification of basal respiration in wild-type and *mpv17^−/−^* mutants (*n*=40). (C) Protein blot analysis of different subunits of OXPHOS complexes. (D) Relative quantification of protein amount, using an antibody against βActin for standardization (*n*=3). R.I., relative intensity. (E) Relative quantification of mtDNA copy number in wild type and *mpv17* homozygous mutants. Mean dCt values were calculated as Ct of *mt-nd1* (mitochondrially encoded gene) minus Ct of *polg* (nuclear gene) and plotted with s.e.m. (*n*=7). (F) Real-time PCR quantification of mRNA transcripts from Ubiquinol-cytochrome c reductase complex subunits (*n*=6). Statistical analyses were performed using two-tailed Student's *t*-test. Statistical significance was evaluated by setting a confidence interval of 95%; data are mean±s.e.m. ****P<0.0001; **P<0.01; *P<0.05; ns, not significant. Experiments were performed in biological replicates.

To understand whether loss of mitochondrial normal morphology in *mpv17* KO mutants was affecting respiratory complexes (RCs), we performed an *in vivo* analysis of the oxygen consumption rate (OCR) in 4 dpf larvae (Fig. 3A). We detected a significant reduction in the basal respiration level in *mpv17^−/−^* mutants compared with wild type (Fig. 3B), thus confirming the impairment of oxidative phosphorylation (OXPHOS) in larvae lacking Mpv17. To enquire whether the RC dysfunction observed was due to a depletion of OXPHOS subunits and complexes, we performed a western blot analysis (Fig. 3C). The cellular amount of all investigated subunits of RC complexes was lower in *mpv17^−/−^* mutants (Fig. 3D); the nuclear-encoded Uqcrc2b, a subunit of complex III, appeared the most affected.

To test whether mtDNA depletion was the cause of the observed reduction in OXPHOS complexes, we assessed mtDNA copy number in *mpv17* homozygous mutants by performing RT-qPCR analysis of DNA extracted from whole larvae. Surprisingly, for a MDS gene, we could not find a significant reduction in mtDNA content in *mpv17*^−/−^ larvae at 6 dpf, a time point at which the morphological and respiratory phenotypes were already noticeable (Fig. 3E). However, in 10 dpf *mpv17*^−/−^ larvae, we did detect a significant decrease in mtDNA content, thus validating the zebrafish *mpv17* KO mutant as a model for *MPV17*-related MDS.

To test whether the OXPHOS reduction was due to defective gene transcription, we investigated, by RT-qPCR, the expression levels of a nuclear- and a mitochondrial-encoded subunit of complex III. No difference was observed in nuclear and mitochondrial transcription when comparing wild-type and *mpv17^−/−^* KO larvae at 6 dpf (Fig. 3F). From these analyses, we can conclude that OXPHOS complex reduction is not due to a defective transcription. Rather, mitochondrial cristae disruption impairs supercomplex localization and functionality (Stuart, 2008), leading to a defective assembly/stability or translation of respiratory complexes.

**Fig. 4.**
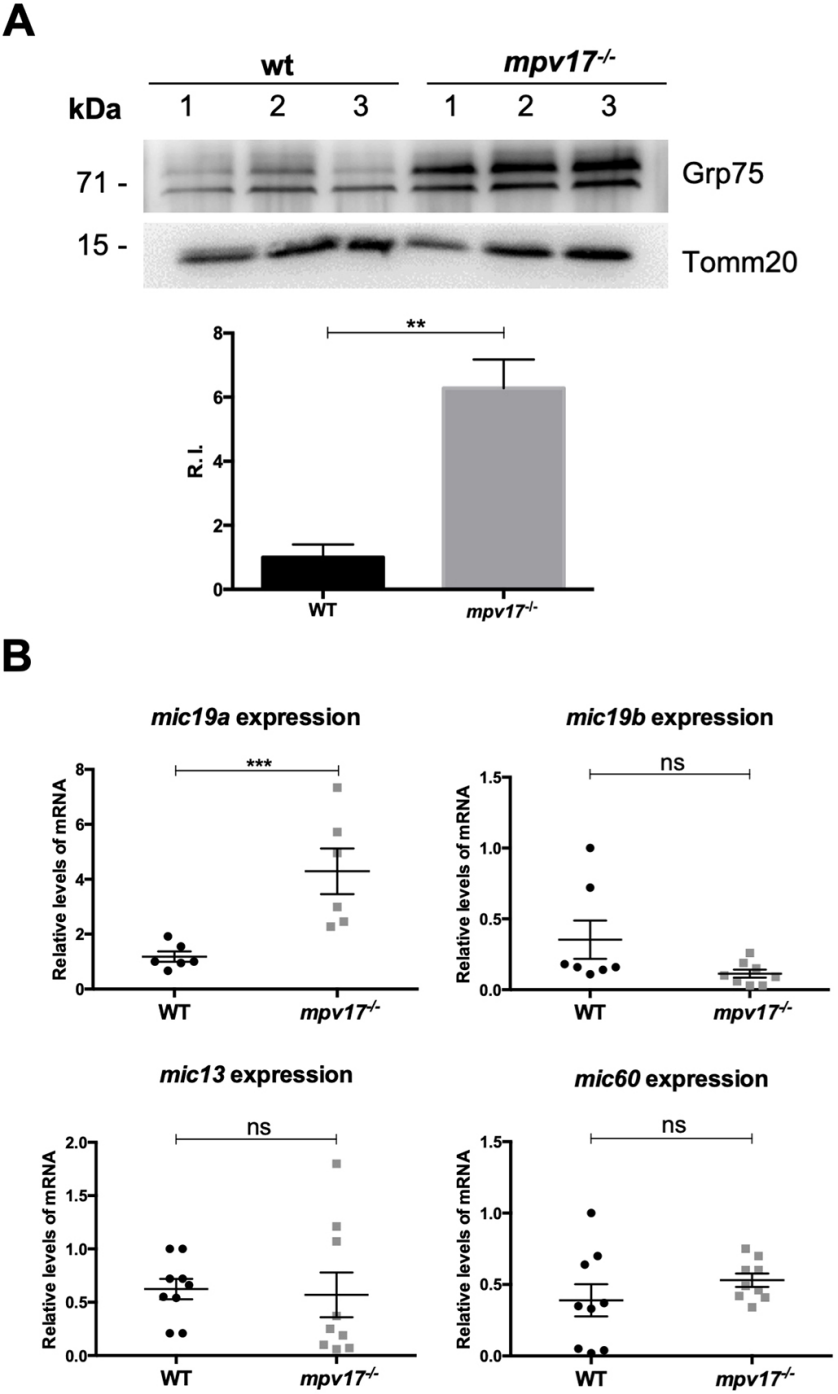
**Evaluation of stress response and mitochondrial quality control system in zebrafish larvae.** (A) Representative western blot analysis of Grp75 protein from 6 dpf zebrafish larvae and relative quantification, using an antibody against Tomm20 (also known as Tomm20b) for standardization (*n*=3). (B) Real-time PCR quantification of mRNA transcripts from different MICOS subunits at 6 dpf (*n*=8). Statistical analyses were performed using two-tailed Student's *t*-test. Statistical significance was evaluated by setting a confidence interval of 95%; data are mean±s.e.m. ***P<0.001; **P<0.01; ns, not significant. Experiments were performed in biological replicates.

Finally, to investigate a possible activation of stress response machinery in *mpv17*^−/−^ mutant mitochondria, we focused on Grp75 (also known as Hspa9), a well-studied cytoprotective factor against oxidative stress (Kaul et al., 2007). Using western blot technique, we found a significant increase in Grp75 in *mpv17* KO mutants compared with wild type at 6 dpf (Fig. 4A). In addition, knowing that the mitochondrial contact site and cristae organizing system (MICOS) acts as a membrane-shaping and -connecting scaffold (Schorr and van der Laan, 2018), we tested whether the loss of mitochondrial ultrastructure might activate the quality control system of the organelle. Thus, we checked, by RT-qPCR, the expression levels of several MICOS subunits: *mic13*, *mic19a*, *mic19b* and *mic60.* As a result, we detected a specific transcriptional upregulation of *mic19a* in *mpv17* KO larvae at 6 dpf (Fig. 4B).

### The pyrimidine *de novo* synthesis pathway is strictly linked to the iridophore phenotype in *mpv17* null mutants

**Fig. 5.**
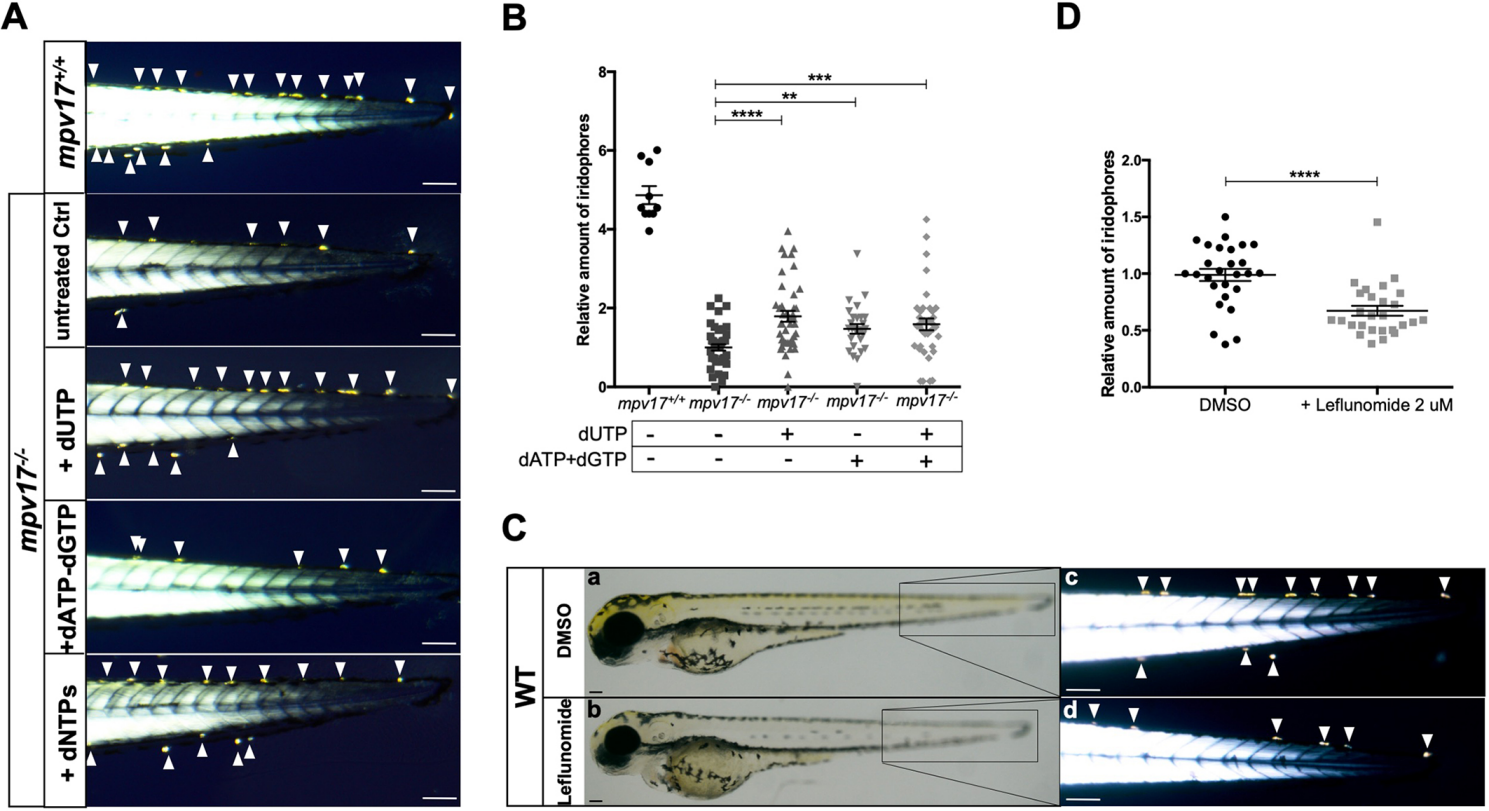
**Investigation of dNTP metabolism in zebrafish larvae.** (A) Visualization of iridophores in the tail region of 3 dpf larvae treated with different mixtures of dNTPs. (B) Relative quantification of iridophore number (*n*=27). (C) Wild-type embryos were treated with dimethyl sulfoxide and 2 μM leflunomide, inhibitor of pyrimidine biosynthesis. Whole-body bright-field pictures (a,b) and birefringent images of the tail region (c,d) are shown. (D) Quantification of relative amounts of iridophores (*n*=27). Arrowheads point to iridophores. Scale bars: 100 µm. Statistical analyses were performed using two-tailed Student's *t*-test. Statistical significance was evaluated by setting a confidence interval of 95%; data are mean±s.e.m. ****P<0.0001; ***P<0.001; **P<0.01. Experiments were performed in biological replicates.

By performing a bioinformatics search on CORD platform to identify *MPV17* co-regulated genes, we found that the trifunctional Carbamoyl-phosphate synthetase 2, aspartate transcarbamylase and dihydroorotase (CAD) enzyme involved in pyrimidine production showed 66.48% of concordance in 176 experiments (Pearson coefficient 0.504 and *P*=1.1×10^−28^), among the highest scores for *MPV17* co-regulated genes (Fig. S3). Remarkably, evidence of a possible connection between pyrimidine *de novo* pathway and the *mpv17* mutant phenotype also comes from previous studies: the inhibition of Dhodh by leflunomide treatment is shown to affect iridophore and melanophore formation in zebrafish (White et al., 2011). In addition, by considering previous studies that suggest a role for Mpv17 in mitochondrial dNTP availability (Löllgen and Weiher, 2014), we decided to treat *mpv17*^−/−^ embryos at 8 h post-fertilization (hpf) with different deoxynucleotide mixtures and to check the amount of iridophores at 3 dpf. Treating the embryos with a solution containing all dNTPs was able to increase iridophore number (Fig. 5A), and dUTP administration had the best effect in ameliorating the *mpv17* KO phenotype (Fig. 5B).

Consequently, we decided to investigate whether the mitochondrial step of pyrimidine *de novo* synthesis was affected in *mpv17* mutants. The conversion of dihydroorotic acid (DHOA) into orotic acid (OA) is a metabolic reaction catalysed by Dhodh, the only mitochondrial enzyme involved in pyrimidine *de novo* synthesis. Hence, we hypothesized that Dhodh functionality might be partially impaired in *mpv17* null mutants. Notably, in accordance with the results by White et al. (2011), we found a specific effect of leflunomide on iridophore formation at low doses; melanophores correctly develop, confirming that inhibition of Dhodh significantly impairs iridophore growth at 3 dpf (Fig. 5C,D).

### OA treatment restores iridophore formation and increases mtDNA copy number in *mpv17* null mutants

**Fig. 6.**
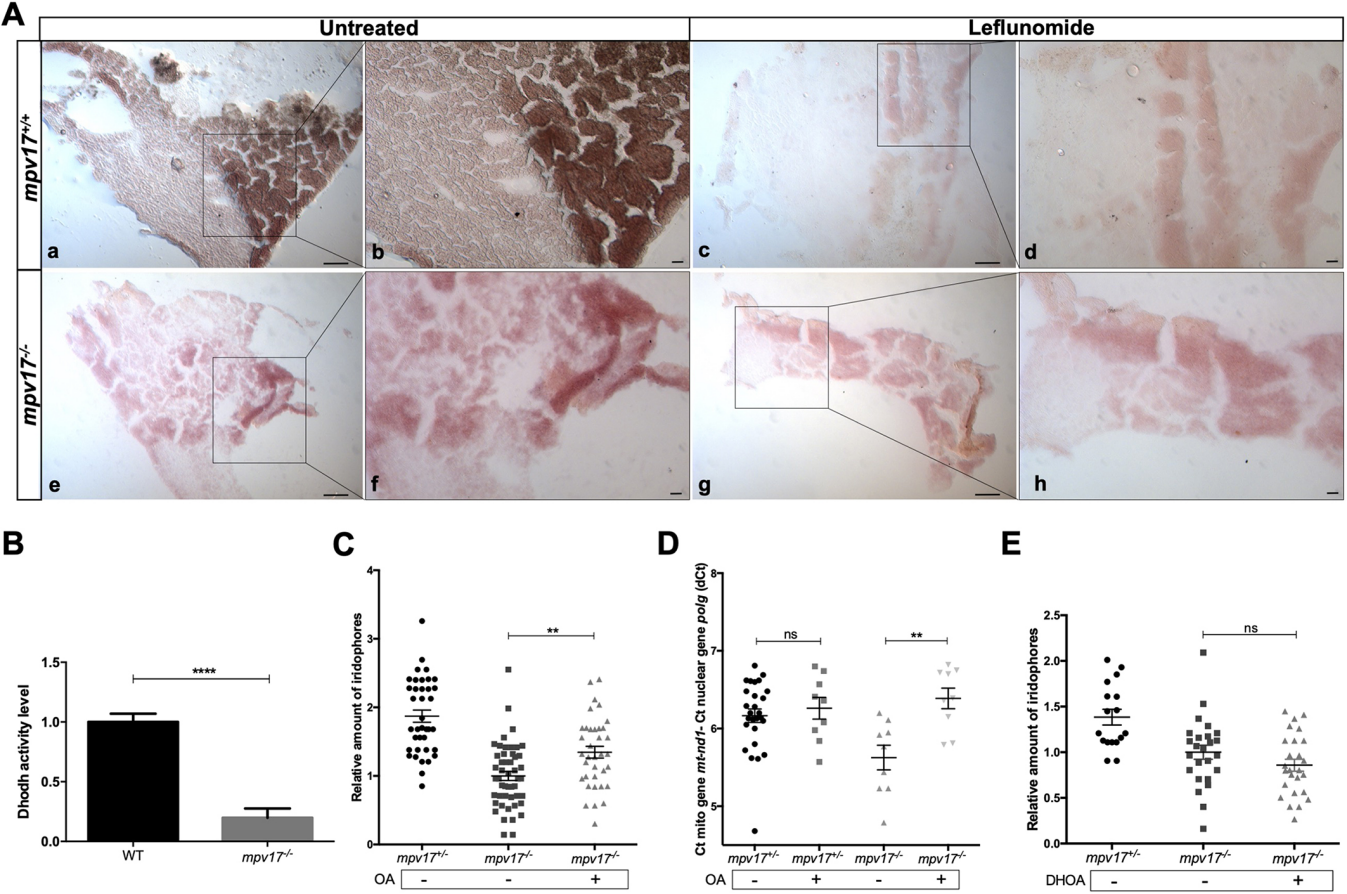
**Analysis of the link between Dhodh functionality and the *mpv17* KO phenotype.** (A) Histochemical Dhodh assay on cryosections of adult wild-type (a-d) and *mpv17* KO (e-h) liver. The activity assay was performed on the same slide and a reaction mix containing 100 μM leflunomide was used as negative control (c,d,g,h). Pictures were taken at lower (a,c,e,g) and higher (b,d,f,h) magnification. Scale bars: 100 µm (*n*=3). (B) Dhodh biochemical assay in 6 dpf larvae. Dhodh was assessed in zebrafish homogenized through spectrophotometric recordings following the reduction of 2,6-dichlorophenolindophenol (DCPIP). Specific Dhodh activity was obtained by subtracting the leflunomide-independent fraction and measured as nmol/min per mg of protein. Values were then normalized with respect to wild-type controls. Statistical analysis was performed using paired Student's *t*-test (*n*=3). (C,D) Relative quantification of iridophore amount in 3 dpf larvae treated with 1 μM orotic acid (OA) (*n*=36) (C) and mtDNA copy number analysis (*n*=8) (D). (E) Relative evaluation of iridophore number in 3 dpf larvae treated with 100 μM dihydroorotic acid (DHOA) (*n*=17). Statistical analyses were performed using two-tailed Student's *t*-test. Statistical significance was evaluated by setting a confidence interval of 95%; data are mean±s.e.m. ****P<0.0001; **P<0.01; ns, not significant. Experiments were performed in biological replicates.

To test a connection between Dhodh impairment and the *mpv17* mutant phenotype, we assessed Dhodh activity by performing a histochemistry assay on liver cryosections of wild-type and *mpv17^−/−^* adults. We decided to perform this analysis on hepatic tissue because Dhodh is highly active in liver, as reported by Löffler et al. (1996), and also because we had found a severe disruption of hepatic mitochondria in *mpv17* KO larvae. Interestingly, we observed significantly lower Dhodh enzymatic activity in *mpv17* null mutants compared with wild type, an activity similar to that of leflunomide-treated samples (Fig. 6A). The biochemical assay confirmed Dhodh impairment in the lysate of *mpv17* KO larvae at 6 dpf (Fig. 6B). In particular, the specific activity of Dhodh in wild type (average value 4534 nmol/min per mg protein), highly similar to that measured in rat liver by Löffler et al. (1996), decreased to 20% in *mpv17* null mutant larvae (average value of 1848 nmol/min per mg protein). These results highlight that the functionality of Dhodh mitochondrial enzyme is reduced at early stages of development and indicate that the impairment of Dhodh enzymatic activity might be the main cause of premature loss of iridophores in *mpv17* null mutants.

In order to examine *in vivo* the mitochondrial reaction of the pyrimidine metabolic pathway, we tested whether administration of OA, the product of Dhodh reaction, might rescue the *mpv17*^−/−^ mutant phenotype (Fig. S4A). When OA was injected into the yolk sac of 20 hpf embryos we could observe a clear rescue of iridophores in *mpv17* null larvae at 3 dpf (Fig. 6C). In addition, while *mpv17* mutants displayed a constant, although non-significant, decrease in mtDNA copy number at 3 dpf, *mpv17*^−/−^ larvae treated with OA exhibited increased mtDNA copy number, with no effect on heterozygotes (Fig. 6D).

Finally, we tested the effect of DHOA (the substrate of Dhodh enzyme) in *mpv17* mutants. As expected, L-DHOA injected at 20 hpf into *mpv17* mutant embryos had no effect on the phenotype, thus confirming Dhodh deficiency in *mpv17* null mutants (Fig. 6E; Fig. S4B).

## DISCUSSION

Our work validates *roy* as a model for investigating the role of *mpv17* and a powerful tool for developing new therapeutic strategies against *MPV17*-related MDS. First, as already reported by D'Agati et al. (2017), we confirmed that zebrafish *mpv17* mRNA can rescue the *mpv17*^−/−^ mutant phenotype, and, most importantly, that human *MPV17* mRNA also has similar properties *in vivo*, thus supporting a conserved function of the two orthologues; this finding is also in agreement with complementation assays in the yeast *sym1*Δ model (Trott and Morano, 2004), showing a functional link between Sym1 and mammalian MPV17. In addition, a recent work demonstrated that the growth of *sym1*Δ strain transformed with R51Q (hR50Q) mutant Sym1-HA allele was impaired (Gilberti et al., 2018). In agreement, also in zebrafish, the injection of the *p.*R50Q mutated form of human *MPV17* mRNA resulted in a mild rescue of the *mpv17*^−/−^ KO phenotype.

Considering that mutants showing minor phenotypical features might exhibit a compensatory upregulation of other members of the gene family (Rossi et al., 2015), we investigated possible feedback mechanisms acted by *mpv17* paralogous genes, also considering that both paralogous proteins (MPV17L and MPV17L2) were previously reported to be mitochondrial proteins (Dalla Rosa et al., 2014; Krick et al., 2008). We could observe increased expression of *mpv17l2* in *mpv17* null mutants. Moreover, overexpression of Mpv17L2 was able to rescue the chromatophore phenotype of *mpv17*^−/−^ mutants. These results support the idea of partial overlapping cellular functions for Mpv17 and Mpv17l2, at least in zebrafish. This finding contrasts with a previous work, carried out in human cellular models, that proposes different roles for MPV17, claimed to be involved in purine synthesis (Dalla Rosa et al., 2016), and MPV17L2, suggested to play a role in mitochondrial ribosome assembly (Dalla Rosa et al., 2014). On the other hand, the tissue-restricted *mpv17l2* overexpression we detected was not able to prevent the strong mitochondrial phenotype assessed in *mpv17* zebrafish mutants. Thus, the role of *mpv17l2* in mitochondria is still an open question. In addition, we did not observe any significant effect of *mpv17l2* mRNA injection on the mtDNA stability of *mpv17* KO larvae. A possible explanation is the lack of significant mtDNA replication, unnecessary at early stages of development (Artuso et al., 2012), thus hampering the evaluation of the role of *mpv17l2* at these time points. Because of that, stable lines overexpressing *mpv17l2* in an *mpv17*^−/−^ mutant background might be useful tools to follow its effect at later stages of development. Moreover, it is important to underline that the injection of *mpv17l2* mRNA leads to non-physiological and ubiquitous expression of Mpv17l2, which is normally not expressed in neural-crest-derived cells, but rather restricted to liver tissue and the digestive system at later stages (Thisse and Thisse, 2004) (Fig. S1). This tissue-specific expression of *mpv17l2* might be the reason why *mpv17* KO mutants, although showing significant overexpression of *mpv17l2*, still lack iridophores, while they do not display any overt hepatic defect at the anatomical level.

In the past two decades, different *MPV17-*related MDS models have been developed, all showing phenotypes that are only partially representative of human clinical features. Whereas the *Mpv17* KO mouse strain mainly shows glomerulosclerosis and inner ear defects (Löllgen and Weiher, 2014), the zebrafish *mpv17* null mutant phenotype is mostly characterized by a specific lack of iridophores and a progressive loss of melanophores, both derived from the neural crest. This phenotype perfectly matches the *Mpv17^−/−^* mouse premature greying (Viscomi et al., 2009) and the progressive demyelinating peripheral neuropathy found in 38% of *MPV17* mutant patients (El-Hattab et al., 2018), features that we can consider possible neurocristopathies.

Notably, in this work, we demonstrate that zebrafish *mpv17* mutant larvae display a severe mitochondrial phenotype, characterized by disappearance of mitochondrial cristae in liver hepatocytes, impairment and reduction of RC complexes, mtDNA depletion and activation of mitochondrial stress response. Alterations of the mitochondrial ultrastructure were previously found in *Mpv17* KO mouse liver (Viscomi et al., 2009) and in a yeast *sym1*Δ model (Dallabona et al., 2010). Moreover, Dallabona and colleagues also suggested that Sym1 is primarily involved in mitochondrial ultrastructure maintenance (Dallabona et al., 2010), taking into account that the loss of cristae architecture affects mtDNA stability and supercomplex arrangement within inner mitochondrial membrane (Stuart, 2008). Our findings strongly support this hypothesis as we were able to observe a significant reduction in mtDNA copy number only at 10 dpf, while mitochondrial ultrastructure and RC complexes were already impaired at 6 dpf. Moreover, a tissue-specific dysfunction in OXPHOS complexes is frequently found in patients affected by different mutations in the *MPV17* gene (Spinazzola et al., 2006*;* Uusimaa et al., 2014; El-Hattab et al., 2018). Taken together, our results prove that the *mpv17* null mutant perfectly resembles the human mitochondrial phenotype, thus indicating that *roy* is a powerful model for *MPV17*-related MDS.

Additionally, the overproduction of Grp75 and the overexpression of *mic19a* we found in *mpv17*^−/−^ mutants highlight the importance of *mpv17* in preserving mitochondrial homeostasis, suggesting that, in the absence of Mpv17, protective mechanisms act against oxidative stress and loss of mitochondrial morphology. Interestingly, the *grp75* yeast orthologue gene, *SSC1*, was also found upregulated at the transcriptional level in the yeast *mpv17* orthologue mutant *sym1*Δ (Trott and Morano, 2004). Moreover, it is indeed well-established that injured mitochondria trigger ROS production via further electron escape from impaired electron transport chain (Maharjan et al., 2014); notably, enhanced ROS levels were previously reported in mice and cell lines depleted of MPV17 (Wagner et al., 2005; Shvetsova et al., 2017).

*MPV17* was discovered about 30 years ago; however, its function remains unknown. To date, the leading hypothesis is that MPV17 may represent an inner mitochondrial membrane protein involved in nucleotide supply for mtDNA synthesis (Löllgen and Weiher, 2014). As for other MDS-related mutations, *MPV17* mutations might impair deoxyribonucleotide metabolism, leading to limited dNTP availability and subsequent mtDNA depletion. Indeed, administration of dNTPs is a therapeutic strategy for treatment of MDS-like mitochondrial neurogastrointestinal encephalopathy (MNGIE) syndrome (Cámara et al., 2014). It was previously reported that supplementation with different amino acids, all precursors in the synthesis of nucleotides, partially restored aerobic oxidative growth in the yeast *sym1*Δ strain (Dallabona et al., 2010). Coherently, we found that deoxyribonucleotide administration strongly increased iridophore number in *mpv17^−/−^* larvae. In particular, because dUTP showed the best effect, we focused on pyrimidine *de novo* synthesis. Serendipitously, by performing a bioinformatics analysis, we found that *MPV17* and *CAD*, a cytosolic enzyme involved in pyrimidine production, are highly co-regulated. Moreover, we observed that a mild inhibition of Dhodh enzyme specifically impaired iridophore development and that Dhodh activity is significantly reduced in *mpv17^−/−^* mutants, in agreement with the well-established link between the Dhodh-mediated mitochondrial step of pyrimidine production and neural crest cell formation (White et al., 2011). Finally, administration of OA, but not DHOA, significantly rescued the phenotype of *mpv17*^−/−^ larvae. Taken all together, our data indicate insufficient pyrimidine synthesis and impairment of Dhodh in *mpv17^−/−^* null mutants, possibly the cause of their main phenotypical trait. Whether this is due to the disruption of mitochondrial cristae, in which the enzyme is located, or to a possible interaction between Dhodh and Mpv17, needs to be further investigated. Most remarkably, the ability of OA (considered in the past vitamin B13) to increase mtDNA content in *mpv17* KO mutants, the availability of this nutrient in dairy products and its current use as a food supplement open new opportunities in the treatment of *MPV17*-related MDS, to date a disease with poor prognosis and no cure yet available.

## MATERIALS AND METHODS

### Zebrafish husbandry and maintenance

All experiments were performed in accordance with the European and Italian Legislations and with permission for animal experimentation from the Local Ethics Committee of the University of Padova. *Danio rerio* (zebrafish) were maintained in a temperature-controlled (28.5°C) environment and fed as described by Kimmel et al. (1995). The *roy* strain was originally obtained from the Harvard Medical School (Boston, USA), and genotyping at early stages was performed by PCR with the following primers: forward, 5′-AACCGTTTGTCATAATGTGGC-3′; reverse-wt, 5′-CATGGGTGTTTGGCCATCAG-3′; reverse-del, 5′-ACATCAACTACATGGGTGGG-3′.

For *in vivo* studies, the following transgenic lines, crossed with *roy*, were used: Tg(*lfabf:dsRed*; *elaA:EGFP*), generated by Korzh et al. (2008); *Tg*(*COXVIII-mls:EGFP*), generated in our laboratory by N.F., using a construct kindly provided by the Seok-Yong laboratory (Chonnam National University Medical School, Gwangju, Republic of Korea), containing the mitochondrial localization sequence of subunit VIII of human cytochrome c oxidase (COXVIII; also known as COX8A).

### RNA extraction and RT-qPCR

Total mRNA was isolated from 50 larvae using Trizol (Invitrogen) and reverse-transcribed using SuperScript^®^ III First-Strand (Thermo Fisher Scientific). Quantitative PCR (qPCR) was performed using a Rotor-gene Q (Qiagen) and 5× HOT FIREPol^®^ EvaGreen*^®^* qPCR Mix Plus (Solis BioDyne), following the manufacturers’ protocols. The cycling parameters were 95°C for 14 min, followed by 45 cycles at 95°C for 15 s, 59°C for 25 s and 72°C for 20 s. Threshold cycles (Ct) and dissociation curves were generated automatically by Rotor-Gene Q series software. Primer sequences are listed in Table 1. Sample Ct values were normalized with Ct values from zebrafish *gapdh*.

**Table 1.**
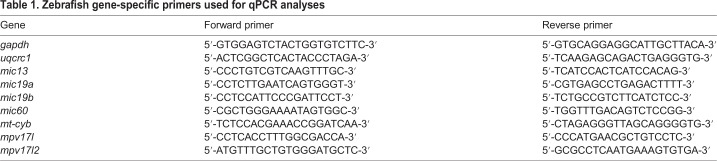
**Zebrafish gene-specific primers used for qPCR analyses**

### Whole-mount ISH

According to Thisse and Thisse (2004), the full-length sequence of *mpv17l2* (ENSDARG00000056367) was PCR amplified from liver complementary DNA (cDNA) of adult zebrafish, using the following primers, designed by Primer3 software (http://primer3.ut.ee): *mpv17l2* probe-F, 5′-CGACTCATGTTGGCTGCTC-3′; *mpv17l2* probe-R, 5′-GCCCAGCGTATGTCACAAAT-3′. The PCR products were purified, then cloned into pCR2.1 TOPO vector (TOPO TA Cloning Dual Promoter Kit, Stratagene) and sequence verified. For riboprobe *in vitro* transcription, the constructs were linearized with the appropriate endonuclease enzyme (Promega) and transcribed with DIG-labelling mix and RNA polymerase (Roche): for antisense (working) riboprobe, *Hind*III/T7 RNApol; for sense (negative control) probe, *Not*I/SP6 RNApol. Whole-mount ISH was performed on zebrafish larvae at 3 dpf, previously fixed with 4% paraformaldehyde (PFA)/PBS and stored in 100% methanol, following standard protocols. At least 20 larvae per condition were processed in a single tube. For signal comparison, wild-type control and *mpv17*^−/−^ mutant larvae were co-processed and co-stained in the same tube; controls were recognized by tail tip excision, performed after PFA fixation and before whole-mount ISH.

### mRNA rescue

The full-length *mpv17* and *mpv17l2* cDNAs from wild-type strain and human *MPV17* cDNA from pCR2.1 TOPO vector, kindly provided by the Zeviani laboratory (University of Cambridge, Cambridge, UK), were subcloned into the pCS2+ vector. The human *MPV17 p.*R50Q (CGG>CAG) mutated form was generated, starting from wild-type construct, using a Q5^®^ Site-Directed Mutagenesis Kit (New England Biolabs) and the following primers: forward, 5′-CAGAGAGGCCAGACTCTGACCATG-3′; reverse, 5′-GTGTTCCTGCAGACCCCG-3′, both designed by NEBaseChanger online tool (New England Biolabs). Capped mRNA was transcribed using the mMessage mMachine^®^ kit (Thermo Fisher Scientific) according to the manufacturer's protocol. The mRNA was resuspended in water and microinjected into one-cell *mpv17*^−/−^ embryos at 70 ng/μl.

### Iridophore count

Iridophore number was measured with the aid of two polarizing filters, basing on the physical property of these cells of being birefringent. Larvae at 3 dpf were anaesthetized with tricaine (0.16 mg/ml), placed on a glass, and the top filter was twisted until it was possible to see the light refracting through the striated muscle. Birefringence analysis was performed according to published methods (Smith et al., 2013). Samples were photographed using a Leica MDG41 microscope and a Nikon Digital Sight DS Fi2 camera; iridophores were manually counted in the tail region and their amount was normalized to that of controls.

### Relative quantification of mtDNA content in whole larvae and sections

DNA samples, extracted from 20 pooled larvae or sections at 3, 6 and 10 dpf, were prepared as described by Rahn et al. (2015) and quantified by PicoGreen assay (Invitrogen) using the Infinite 200 PRO plate reader (Tecan). Then, 8.5 ng of each sample was used in a qPCR reaction and run in triplicate, using 5× HOT FIREPol^®^ EvaGreen^®^ qPCR Supermix (Solis Biodyne). mtDNA copy number was determined by the RT-qPCR standard curve method using *mt-nd1* as the mitochondrial gene target and the *polg* gene as a reference for nDNA, following the protocol developed by Artuso et al. (2012). The delta Ct (dCt) value was calculated by subtracting the Ct of *polg* from the Ct of *mt-nd1* for each sample.

### TEM analysis

Larvae were anaesthetized and fixed with 2.5% glutaraldehyde in 0.1 M sodium cacodylate buffer, then dehydrated, embedded in epoxy resin and sectioned following a standard TEM sample preparation protocol. All steps after fixation were performed by the TEM service of the Department of Biology (University of Padova).

### Confocal analysis and mitochondrial volume measurements

Double transgenic fluorescence of Tg(*lfabf:dsRed*; *COXVIII-mls:EGFP*) in *mpv17*^−/−^ mutant background was visualized under a Leica M165FC dissecting microscope and then with a Nikon C2 H600 L confocal microscope. Larvae were anaesthetized and mounted in 1% low-melting-point agarose gel. Mitochondrially targeted EGFP and cytosolic dsRed fluorescence was visualized by using 488 nm and 561 nm lasers, through a 40× immersion objective (Nikon). All images were analysed with Fiji software (https://fiji.sc/). Mitochondria were automatically segmented on the EGFP fluorescence image stacks, inside a dsRed fluorescent mask identifying hepatic tissue, and total mitochondrial volume was calculated, according to previous work (Facchinello et al., 2016). After confocal acquisition, heterozygous and homozygous siblings were genotyped by PCR on DNA previously extracted from a single larva, as described by Gagnon et al. (2014).

### Measurement of OCR

OCR was measured in zebrafish larvae at 96 hpf with a Seahorse XF24 extracellular flux analyser. Larvae were placed into the XF24 microplate well (one larva per well) and blocked with a capture screen in the presence of 670 μl fish water (0.5 mM NaH_2_PO_4_, 0.5 mM NaHPO_4_, 3 mg/l instant ocean). Basal respiration was measured for 63 min at 28.5°C, while the maximal respiration was measured upon administration of 0.5 μM carbonyl cyanide-4-phenylhydrazone (FCCP). A mixture of 2 μM rotenone (Rot) and 5 μM antimycin A was added to shut down electron transport chain function, revealing the non-mitochondrial respiration. Basal respiration was obtained by subtracting non-mitochondrial respiration. Respiratory rates are the average±s.e.m. of at least 20 individual larvae per condition.

### Western blot analysis

Proteins were extracted from 30 pooled larvae at 6 dpf. Lysis was performed in ice-cold RIPA buffer [125 mM NaCl, 25 mM Tris-HCl pH 7.4, 1 mM EGTA-Tris pH 7.4, 1% Triton X-100, 0.5% sodium deoxycholate, 0.1% SDS and complete EDTA-free protease inhibitor cocktail (Roche)]. Crude lysate was cleared by centrifuging for 30 min at 15,000 ***g*** and proteins in the supernatant were quantified using a BCA Protein Assay Kit (Pierce). Equal amounts of proteins (50 μg) were loaded on 4-12% Bis-Tris NuPage gels (Life Technologies) and blotted on PVDF Immobilon-P membranes (Millipore). Dried membranes were then washed with TBS buffer (50 mM Tris-HCl pH 7.5, 50 mM NaCl) with 1% (w/v) bovine serum albumin (BSA; Sigma-Aldrich) and incubated overnight with the indicated primary antibodies at 4°C: Mitoprofile^®^ Human WB Total OXPHOS cocktail antibody (1:100; Abcam, ab110411), anti-Grp75 antibody (1:1000; Santa Cruz Biotechnology, H-155), anti-Actin antibody (1:1000; Santa Cruz Biotechnology, AC-15) or anti-Tomm20 antibody (1:10,000; Sigma-Aldrich, HPA011562). Secondary horseradish-peroxidase-conjugated antibodies (Bio-Rad) were incubated for 1 h at room temperature and protein bands were detected by chemiluminescence on a UVITEC Alliance Mini HD9. Quantitation of the signal was performed with ImageJ software (https://imagej.nih.gov/ij/).

### Compound administration

Deoxynucleotide Set (SLBH8759), OA (O2750) and L-DHOA (D7128) were obtained from Sigma-Aldrich. dNTPs (12.5 µM each) were added to embryo medium at 8 hpf, and OA (1 µM) and L-DHOA (100 µM) were injected into the yolk sac of 20 hpf embryos as described by Willer et al. (2005). Controls were injected at 20 hpf, with a control solution containing NaOH or dimethylformamide, and phenotypic rescue was evaluated at 3 dpf.

### Dhodh activity assays

To measure Dhodh activity, 6 dpf larvae were homogenized in a buffer composed of 100 mM HEPES pH 7.5, 150 mM NaCl, 0.01% Triton X-100, protease and phosphatase inhibitors. Cell homogenates (60 μg) were pre-incubated for 10 min with the substrate L-DHOA (10 mM) and alamethicin (2 mM), and separately with the Dhodh inhibitor leflunomide (200 μM). After the pre-incubation time, sodium azide (250 mM), antimycin A (2 mM), rotenone (2 mM), 3-nitropropionic acid (2 mM), Coenzyme Q_1_ (10 mM) and 2,6-dichlorophenolindophenol (DCPIP; 5 mM) were added. Dhodh activity was assessed at 30°C through spectrophotometric recordings following the reduction of DCPIP at 600 nM (ε=19.1×mM^−1^×cm^−1^). Values were normalized for protein amount.

Dhodh enzymatic activity was also histochemically assessed on frozen tissue. The sections were incubated for 30 min at 37°C with a medium containing HEPES (150 mM) pH 8, L-DHOA (10 mM), sodium azide (1 mM) and Nitro Blue Tetrazolium (NBT) (1 mM). The Dhodh inhibitor leflunomide (200 µm) was included in the test. Successively, the sections were rinsed with a wash solution containing HEPES (150 mM) pH 8 and water and covered using a mounting medium. The DHOA was oxidized to OA, producing FMNH_2_, and the reaction was visualized by chemically reacting the electron acceptor with the purple salt NBT. Samples were photographed using a Leica DMR microscope equipped with a Leica DFC7000T digital camera.

### Statistics

Prism software (GraphPad) was used for data analysis and figure plotting. All experiments were performed at least in biological triplicate. Standard error of the mean calculations and all statistical analyses were performed using Student's *t*-test.

## Supplementary Material

Supplementary information

## References

[DMM037226C1] Artuso L., Romano A., Verri T., Domenichini A., Argenton F., Santorelli F. M. and Petruzzella V. (2012). Mitochondrial DNA metabolism in early development of zebrafish (*Danio rerio*). *Biochim. Biophys. Acta* 1817, 1002-1011. 10.1016/j.bbabio.2012.03.019 22465854

[DMM037226C2] Calvo S. E., Clauser K. R. and Mootha V. K. (2016). MitoCarta2.0: an updated inventory of mammalian mitochondrial proteins. *Nucleic Acids Res.* 44, D1251-D1257. 10.1093/nar/gkv1003 26450961 PMC4702768

[DMM037226C3] Cámara Y., González-Vioque E., Scarpelli M., Torres-Torronteras J., Caballero A., Hirano M. and Martí R. (2014). Administration of deoxyribonucleosides or inhibition of their catabolism as a pharmacological approach for mitochondrial DNA depletion syndrome. *Hum. Mol. Genet.* 23, 2459-2467. 10.1093/hmg/ddt641 24362886 PMC6281351

[DMM037226C4] Chinnery P. F. and Hudson G. (2013). Mitochondrial genetics. *Br. Med. Bull.* 106, 135-159. 10.1093/bmb/ldt017 23704099 PMC3675899

[DMM037226C5] D'Agati G., Beltre R., Sessa A., Burger A., Zhou Y., Mosimann C. and White R. M. (2017). A defect in the mitochondrial protein Mpv17 underlies the transparent casper zebrafish. *Dev. Biol.* 430, 11-17. 10.1016/j.ydbio.2017.07.017 28760346 PMC5617342

[DMM037226C6] Dallabona C., Marsano R. M., Arzuffi P., Ghezzi D., Mancini P., Zeviani M., Ferrero I. and Donnini C. (2010). Sym1, the yeast ortholog of the MPV17 human disease protein, is a stress-induced bioenergetic and morphogenetic mitochondrial modulator. *Hum. Mol. Genet.* 19, 1098-1107. 10.1093/hmg/ddp581 20042463

[DMM037226C7] Dalla Rosa I., Durigon R., Pearce S. F., Rorbach J., Hirst E. M. A., Vidoni S., Reyes A., Brea-Calvo G., Minczuk M., Woellhaf M. W. et al. (2014). MPV17L2 is required for ribosome assembly in mitochondria. *Nucleic Acids Res.* 42, 8500-8515. 10.1093/nar/gku513 24948607 PMC4117752

[DMM037226C8] Dalla Rosa I., Cámara Y., Durigon R., Moss C. F., Vidoni S., Akman G., Hunt L., Johnson M. A., Grocott S., Wang L. et al. (2016). MPV17 Loss Causes Deoxynucleotide Insufficiency and Slow DNA Replication in Mitochondria. *PLoS Genet.* 12: e1005779 10.1371/journal.pgen.1005779 26760297 PMC4711891

[DMM037226C9] El-Hattab A. W., Wang J., Dai H., Almannai M., Staufner C., Alfadhel M., Gambello M. J., Prasun P., Raza S., Lyons H. J. et al. (2018). MPV17-related mitochondrial DNA maintenance defect: new cases and review of clinical, biochemical, and molecular aspects. *Hum. Mut.* 39, 461-470. 10.1002/humu.23387 29282788

[DMM037226C10] Facchinello N., Schiavone M., Vettori A., Argenton F. and Tiso N. (2016). Monitoring Wnt Signaling in Zebrafish Using Fluorescent Biosensors. *Methods Mol. Biol.* 1481, 81-94. 10.1007/978-1-4939-6393-5_9 27590154

[DMM037226C11] Frohnhöfer H. G., Krauss J., Maischein H. M. and Nüsslein-Volhard C. (2013). Iridophores and their interactions with other chromatophores are required for stripe formation in zebrafish. *Development* 140, 2997-3007. 10.1242/dev.096719 23821036 PMC3912879

[DMM037226C12] Gagnon J. A., Valen E., Thyme S. B., Huang P., Akhmetova L., Pauli A., Montague T. G., Zimmerman S., Richter C. and Schier A. F. (2014). Efficient mutagenesis by Cas9 protein-mediated oligonucleotide insertion and large-scale assessment of single-guide RNAs. *PLoS ONE* 9, e98186 10.1371/journal.pone.0098186 24873830 PMC4038517

[DMM037226C13] Gilberti M., Baruffini E., Donnini C. and Dallabona C. (2018). Pathological alleles of MPV17 modeled in the yeast Saccharomyces cerevisiae orthologous gene SYM1 reveal their inability to take part in a high molecular weight complex. *PLoS ONE* 13, e0205014 10.1371/journal.pone.0205014 30273399 PMC6166979

[DMM037226C14] Higdon C. W., Mitra R. D. and Johnson S. L. (2013). Gene expression analysis of zebrafish melanocytes, iridophores, and retinal pigmented epithelium reveals indicators of biological function and developmental origin. *PLoS ONE* 8, e67801 10.1371/journal.pone.0067801 23874447 PMC3706446

[DMM037226C15] Karadimas C. L., Vu T. H., Holve S. A., Chronopoulou P., Quinzii C., Johnsen S. D., Kurth J., Eggers E., Palenzuela L., Tanji K. et al. (2006). Navajo neurohepatopathy is caused by a mutation in the MPV17 gene. *Am. J. Hum. Genet.* 79, 544-548. 10.1086/506913 16909392 PMC1559552

[DMM037226C16] Kaul S. C., Deocaris C. C. and Wadhwa R. (2007). Three faces of mortalin: a housekeeper, guardian and killer. *Exp. Gerontol.* 42, 263-274. 10.1016/j.exger.2006.10.020 17188442

[DMM037226C17] Kelsh R. N., Brand M., Jiang Y. J., Heisenberg C. P., Lin S., Haffter P., Odenthal J., Mullins M. C., van Eeden F. J., Furutani-Seiki M. et al. (1996). Zebrafish pigmentation mutations and the processes of neural crest development. *Development* 123, 369-389. 9007256 10.1242/dev.123.1.369

[DMM037226C18] Kelsh R. N., Sosa K. C., Owen J. P., Yates C. A. (2017). Zebrafish adult pigment stem cells are multipotent and form pigment cells by a progressive fate restriction process: clonal analysis identifies shared origin of all pigment cell types. *BioEssays* 39, 1600234 10.1002/bies.201600234 28009049

[DMM037226C19] Kim J., Kang E., Kim Y., Kim J. M., Lee B. H., Murayama K., Kim G. H., Choi I. H., Kim K. M. and Yoo H. W. (2016). MPV17 mutations in patients with hepatocerebral mitochondrial DNA depletion syndrome. *Mol. Genet. Metab. Rep.* 8, 74-76. 10.1016/j.ymgmr.2016.06.006 27536553 PMC4976613

[DMM037226C20] Kimmel C. B., Ballard W. W., Kimmel S. R., Ullmann B. and Schilling T. F. (1995). Stages of embryonic development of the zebrafish. *Dev. Dyn.* 203, 253-310. 10.1002/aja.1002030302 8589427

[DMM037226C21] Korzh S., Pan X., Garcia-Lecea M., Winata C. L., Pan X., Wohland T., Korzh V. and Gong Z. (2008). Requirement of vasculogenesis and blood circulation in late stages of liver growth in zebrafish. *BMC Dev. Biol.* 8, 84 10.1186/1471-213X-8-84 18796162 PMC2564926

[DMM037226C22] Krauss J., Astrinidis P., Frohnhöfer H. G., Walderich B. and Nüsslein-Volhard C. (2013). transparent, a gene affecting stripe formation in Zebrafish, encodes the mitochondrial protein Mpv17 that is required for iridophore survival. *Biol. Open* 2, 703-710. 10.1242/bio.20135132 23862018 PMC3711038

[DMM037226C23] Krick S., Shi S., Ju W., Faul C., Tsai S., Mundel P. and Böttinger E. P. (2008). Mpv17l protects against mitochondrial oxidative stress and apoptosis by activation of Omi/HtrA2 protease. *Proc. Natl. Acad. Sci. USA* 105, 14106-14111. 18772386 10.1073/pnas.0801146105 PMC2529330

[DMM037226C24] Löffler M., Becker C. and Schuster E. W. G. (1996). Catalytic enzyme histochemistry and biochemical analysis of dihydroorotate dehydrogenase/oxidase and succinate dehydrogenase in mammalian tissues, cells and mitochondria. *Histochem. Cell Biol.* 105:t119-t28. 10.1007/BF01696151 8852433

[DMM037226C25] Löllgen S. and Weiher H. (2014). The role of the Mpv17 protein mutations of which cause mitochondrial DNA depletion syndrome (MDDS): lessons from homologs in different species. *J. Biol. Chem.* 396, 13-25. 10.1515/hsz-2014-0198 25205723

[DMM037226C26] Maharjan S., Oku M., Tsuda M., Hoseki J. and Sakai Y. (2014). Mitochondrial impairment triggers cytosolic oxidative stress and cell death following proteasome inhibition. *Sci. Rep.* 4, 5896 10.1038/srep05896 25077633 PMC4116626

[DMM037226C27] Moraes C. T., Shanske S., Tritschler H. J., Aprille J. R., Andreetta F., Bonilla E., Schon E. A. and DiMauro S. (1991). mtDNA depletion with variable tissue expression: a novel genetic abnormality in mitochondrial diseases. *Am. J. Hum. Genet.* 48, 492-501. 1998336 PMC1682992

[DMM037226C28] Ng A., Uribe R. A., Yieh L., Nuckels R. and Gross J. M. (2009). Zebrafish mutations in gart and paics identify crucial roles for de novo purine synthesis in vertebrate pigmentation and ocular development. *Development* 136, 2601-2611. 10.1242/dev.038315 19570845 PMC2709066

[DMM037226C29] Nogueira C., Almeida L. S., Nesti C., Pezzini I., Videira A., Vilarinho L. and Santorelli F. M. (2014). Syndromes associated with mitochondrial DNA depletion. *Ital. J. Pediatr.* 40, 34 10.1186/1824-7288-40-34 24708634 PMC3985578

[DMM037226C30] Rahn J. J., Bestman J. E., Stackley K. D. and Chan S. S. L. (2015). Zebrafish lacking functional DNA polymerase gamma survive to juvenile stage, despite rapid and sustained mitochondrial DNA depletion, altered energetics and growth. *Nucleic Acids Res.* 43, 10338-10352. 10.1093/nar/gkv1139 26519465 PMC4666367

[DMM037226C31] Rossi A., Kontarakis Z., Gerri C., Nolte H., Hölper S., Krüger M. and Stainier D. Y. R. (2015). Genetic compensation induced by deleterious mutations but not gene knockdowns. *Nature* 524, 230-233. 10.1038/nature14580 26168398

[DMM037226C32] Schorr S. and van der Laan M. (2018). Integrative functions of the mitochondrial contact site and cristae organizing system. Semin. *Cell. Dev. Biol.* 76, 191-200. 10.1016/j.semcdb.2017.09.021 28923515

[DMM037226C33] Shvetsova A. N., Mennerich D., Kerätär J. M., Kalervo Hiltunen J. and Kietzmann T. (2017). Non-electron transfer chain mitochondrial defects differently regulate HIF-1α degradation and transcription. *Redox Biol.* 12, 1052-1061. 10.1016/j.redox.2017.05.003 28531964 PMC5440747

[DMM037226C34] Smith L. L., Beggs A. H. and Gupta V. A. (2013). Analysis of skeletal muscle defects in larval zebrafish by birefringence and touch-evoke escape response assays. *J. Vis. Exp.* 13, e50925. 10.3791/50925 PMC4048356 24378748

[DMM037226C35] Spinazzola A., Viscomi C., Fernandez-Vizarra E., Carrara F., D'Adamo P., Calvo S., Marsano R. M., Donnini C., Weiher H., Strisciuglio P. et al. (2006). MPV17 encodes an inner mitochondrial membrane protein and is mutated in infantile hepatic mitochondrial DNA depletion. *Nat. Genet.* 38, 570-575. 16582910 10.1038/ng1765

[DMM037226C36] Stuart R. A. (2008). Supercomplex organization of the oxidative phosphorylation enzymes in yeast mitochondria. *J. Bioenerg. Biomembr.* 40, 411 10.1007/s10863-008-9168-4 18839289

[DMM037226C37] Suomalainen A. and Isohanni P. (2010). Mitochondrial DNA depletion syndromes--many genes, common mechanisms. *Neuromuscul. Disord.* 20, 429-437. 10.1016/j.nmd.2010.03.017 20444604

[DMM037226C38] Thisse B. and Thisse C. (2004). Fast Release Clones: A High Throughput Expression Analysis. ZFIN Direct Data Submission. (http://zfin.org).

[DMM037226C39] Trott A. and Morano K. A. (2004). SYM1 is the stress-induced Saccharomyces cerevisiae ortholog of the mammalian kidney disease gene Mpv17 and is required for ethanol metabolism and tolerance during heat shock. *Eukaryot. Cell.* 3, 620-631. 10.1128/EC.3.3.620-631.2004 15189984 PMC420134

[DMM037226C40] Uusimaa J., Evans J., Smith C., Butterworth A., Craig K., Ashley N., Liao C., Carver J., Diot A., Macleod L. et al. (2014). Clinical, biochemical, cellular and molecular characterization of mitochondrial DNA depletion syndrome due to novel mutations in the MPV17 gene. *Eur. J. Hum. Genet.* 22, 184-191. 10.1038/ejhg.2013.112 23714749 PMC3895632

[DMM037226C41] Viscomi C., Spinazzola A., Maggioni M., Fernandez-Vizarra E., Massa V., Pagano C., Vettor R., Mora M. and Zeviani M. (2009). Early-onset liver mtDNA depletion and late-onset proteinuric nephropathy in Mpv17 knockout mice. *Hum. Mol. Genet.* 18, 12-26. 10.1093/hmg/ddn309 18818194 PMC2644642

[DMM037226C42] Wagner G., Stettmaier K. and Bors W. (2005). Enhanced gamma-glutamyl transpeptidase expression and superoxide production in Mpv17^−/−^ Glomerulosclerosis Mice. *J. Biol. Chem.* 382, 1019-1025. 10.1515/BC.2001.128 11530932

[DMM037226C45] Wallace D. C. (2005). A mitochondrial paradigm of metabolic and degenerative diseases, aging, and cancer: a dawn for evolutionary medicine. *Annu. Rev. Genet*. **39**, 359-407 10.1146/annurev.genet.39.110304.095751 PMC2821041 16285865

[DMM037226C43] White R. M., Cech J., Ratanasirintrawoot S., Lin C. Y., Rahl P. B., Burke C. J., Langdon E., Tomlinson M. L., Mosher J., Kaufman C. et al. (2011). DHODH modulates transcriptional elongation in the neural crest and melanoma. *Nature* 471, 518-522. 10.1038/nature09882 21430780 PMC3759979

[DMM037226C44] Willer G. B., Lee V. M., Gregg R. G. and Link B. A. (2005). Analysis of the Zebrafish perplexed mutation reveals tissue-specific roles for de novo pyrimidine synthesis during development. *Genetics* 170, 1827-1837. 10.1534/genetics.105.041608 15937129 PMC1449754

