# Supplementary material for: The zebrafish orthologue of the human hepatocerebral disease gene MPV17 plays pleiotropic roles in mitochondria
Source: Dis Model Mech. 2019 Mar 14;12(3):dmm037226. doi: 10.1242/dmm.037226 (PMC6451431; doi:10.1242/dmm.037226)
Supplement: Supplementary information [file dmm-12-037226-s1.pdf]

Appendix A. Supplementary information

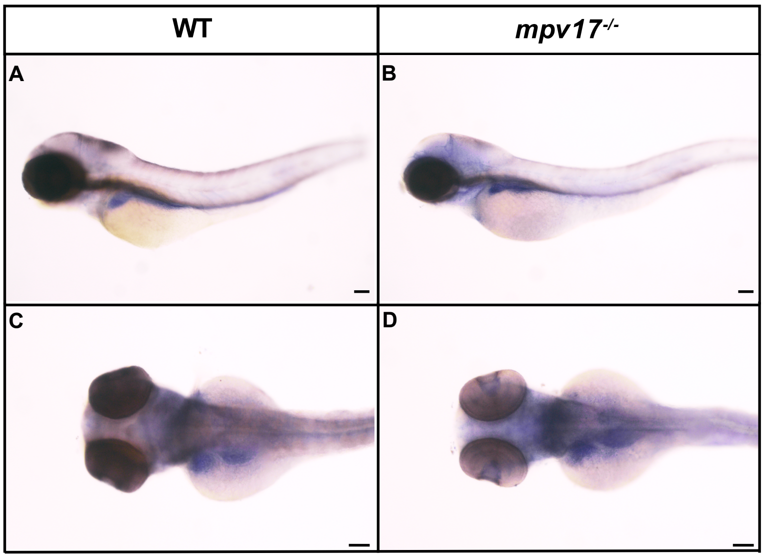

Figure S1. Expression analysis of zebrafish *mpv17-l2* gene in wild-type and *mpv17*$^{-/-}$ mutant larvae at 3 dpf. Lateral (A-B) and dorsal (C-D) view of WISH with anti-*mpv17l2* probe on 3 dpf wild-type and *mpv17*$^{-/-}$ larvae. Bars, 100 µm.

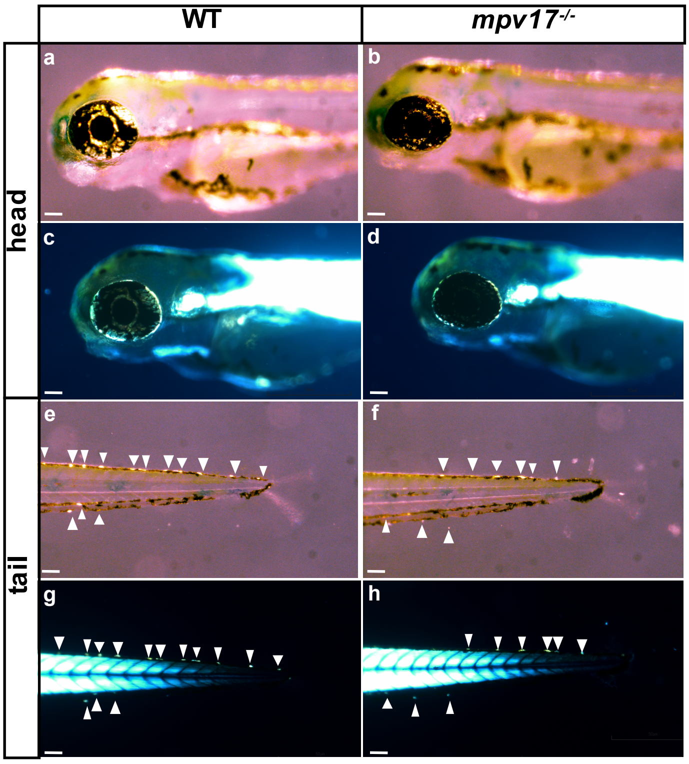

Figure S2. Comparison between different counting methods of iridophores in wild-type and *mpv17*$^{-/-}$ larvae at 3 dpf. Bright-field pictures (a, b, e, f), acquired according to Krauss and colleagues' method: the angle of illumination had been adjusted individually to allow optimal visualization of iridophores (Krauss *et al.*, 2013). Birefringence images (c, d, g, h), acquired by using a polarizer and an analyser lens, according to previously published methods (Smith *et al.*, 2013). Representative images of iridophores along the tail (g, h) or off the eye (c, d) were taken without the need of modifying the incident light and by completely abolishing background. The arrows point to iridophores. Bars, 100 µm.

Disease Models & Mechanisms • Supplementary information

Gene symbol	Description	# of experiments with MPV17	%concordant	Pearson Coef	Pearson p-val.
MPV17	MpV17 mitochondrial inner membrane protein [Source:HGNC Symbol;Acc:7224]	424			
MAGED1	melanoma antigen family D, 1 [Source:HGNC Symbol;Acc:6813]	176	88.6363636364	0.519030597265	1.28356075802e-30
GLB1	galactosidase, beta 1 [Source:HGNC Symbol;Acc:4298]	149	91.9463087248	0.510492925155	1.62691766014e-29
PGAM1	phosphoglycerate mutase 1 (brain) [Source:HGNC Symbol;Acc:8888]	172	87.7906976744	0.508691604107	2.75498214289e-29
CAD	carbamoyl-phosphate synthetase 2, aspartate transcarbamylase, and dihydroorotase [Source:HGNC Symbol;Acc:1424]	176	66.4772727273	0.503918624571	1.0957433225e-28
CNDP2	CNDP dipeptidase 2 (metallopeptidase M20 family) [Source:HGNC Symbol;Acc:24437]	175	84.0	0.503598549838	1.20110287317e-28
YWHAB	tyrosine 3-monooxygenase/tryptophan 5-monooxygenase activation protein, beta polypeptide [Source:HGNC Symbol;Acc:12849]	191	91.0994764398	0.49560445747	1.15309824315e-27
FUCA2	fucosidase, alpha-L- 2, plasma [Source:HGNC Symbol;Acc:4008]	150	92.6666666667	0.490884702241	4.26364953099e-27
FANCF	Fanconi anemia, complementation group F [Source:HGNC Symbol;Acc:3587]	100	89.0	0.486093506765	1.57520696874e-26
GBA	glucosidase, beta, acid [Source:HGNC Symbol;Acc:4177]	150	87.3333333333	0.485906687821	1.65686485517e-26
IDH3G	isocitrate dehydrogenase 3 (NAD+) gamma [Source:HGNC Symbol;Acc:5386]	139	90.6474820144	0.482629959765	4.0002403426e-26
HEXB	hexosaminidase B (beta polypeptide) [Source:HGNC Symbol;Acc:4879]	165	85.4545454545	0.477602077649	1.51878235204e-25
TBL1X	transducin (beta)-like 1X-linked [Source:HGNC Symbol;Acc:11585]	162	75.9259259259	0.468468216355	1.62131042545e-24
CD81	CD81 molecule [Source:HGNC Symbol;Acc:1701]	180	91.6666666667	0.467042697849	2.33127614232e-24
CTSD	cathepsin D [Source:HGNC Symbol;Acc:2529]	173	91.9075144509	0.463243470992	6.08646420812e-24
MCFD2	multiple coagulation factor deficiency 2 [Source:HGNC Symbol;Acc:18451]	197	89.3401015228	0.462693462979	6.98665953951e-24
CTSA	cathepsin A [Source:HGNC Symbol;Acc:9251]	114	90.350877193	0.461043508314	1.05517852598e-23
NME2	NME/NM23 nucleoside diphosphate kinase 2 [Source:HGNC Symbol;Acc:7850]	118	91.5254237288	0.455438251657	4.21115002682e-23
PPIB	peptidylprolyl isomerase B (cyclophilin B) [Source:HGNC Symbol;Acc:9255]	155	88.3870967742	0.454083035203	5.86228108546e-23
SLC38A10	solute carrier family 38, member 10 [Source:HGNC Symbol;Acc:28237]	123	95.1219512195	0.451567966058	1.0789274261e-22
SERPINH1	serpin peptidase inhibitor, clade H (heat shock protein 47), member 1, (collagen binding protein 1) [Source:HGNC Symbol;Acc:1546]	164	92.0731707317	0.446791749227	3.38908757451e-22
PPP1CA	protein phosphatase 1, catalytic subunit, alpha isozyme [Source:HGNC Symbol;Acc:9281]	125	87.2	0.44510334377	5.05752314664e-22
PLOD1	procollagen-lysine, 2-oxoglutarate 5-dioxygenase 1 [Source:HGNC Symbol;Acc:9081]	129	95.3488372093	0.441833289083	1.09120863073e-21
DBP	D site of albumin promoter (albumin D-box) binding protein [Source:HGNC Symbol;Acc:2697]	207	83.0917874396	0.440205260855	1.59527489813e-21
TSEN15	tRNA splicing endonuclease 15 homolog (S. cerevisiae) [Source:HGNC Symbol;Acc:16791]	60	96.6666666667	0.437965250784	2.68109323433e-21
GNS	glucosamine (N-acetyl)-6-sulfatase [Source:HGNC Symbol;Acc:4422]	190	80.5263157895	0.437951472304	2.68963699002e-21
CRTAP	cartilage associated protein [Source:HGNC Symbol;Acc:2379]	190	89.4736842105	0.43732697844	3.10637459412e-21
GLG1	golgi glycoprotein 1 [Source:HGNC Symbol;Acc:4316]	113	92.9203539823	0.433118049145	8.13825635339e-21
BACE1	beta-site APP-cleaving enzyme 1 [Source:HGNC Symbol;Acc:933]	198	89.898989899	0.429986746168	1.65175956867e-20
ALG5	asparagine-linked glycosylation 5, dolichyl-phosphate beta-glucosyltransferase homolog (S. cerevisiae) [Source:HGNC Symbol;Acc:20266]	116	82.7586206897	0.422905015426	7.97073966727e-20
NEU1	sialidase 1 (lysosomal sialidase) [Source:HGNC Symbol;Acc:7758]	150	82.6666666667	0.417421141773	2.62930547926e-19
ZFP1	zinc finger protein 1 homolog (mouse) [Source:HGNC Symbol;Acc:23328]	55	87.2727272727	0.416638528548	3.11202428193e-19
GSTO1	glutathione S-transferase omega 1 [Source:HGNC Symbol;Acc:13312]	176	86.3636363636	0.415561586846	3.92158880601e-19
TSEN34	tRNA splicing endonuclease 34 homolog (S. cerevisiae) [Source:HGNC Symbol;Acc:15506]	54	100.0	0.415357065956	4.09724242333e-19
HEXA	hexosaminidase A (alpha polypeptide) [Source:HGNC Symbol;Acc:4878]	140	84.2857142857	0.414082359695	5.38025750368e-19
GSS	glutathione synthetase [Source:HGNC Symbol;Acc:4624]	121	87.6033057851	0.413013651004	6.75470049517e-19

Figure S3. Bioinformatics search on CORD platform to identify *MPV17* co-regulated genes. The table shows a list of *MPV17* co-regulated genes based on the number of experiments, the percentage of concordance, Pearson coefficient and Pearson p-value.

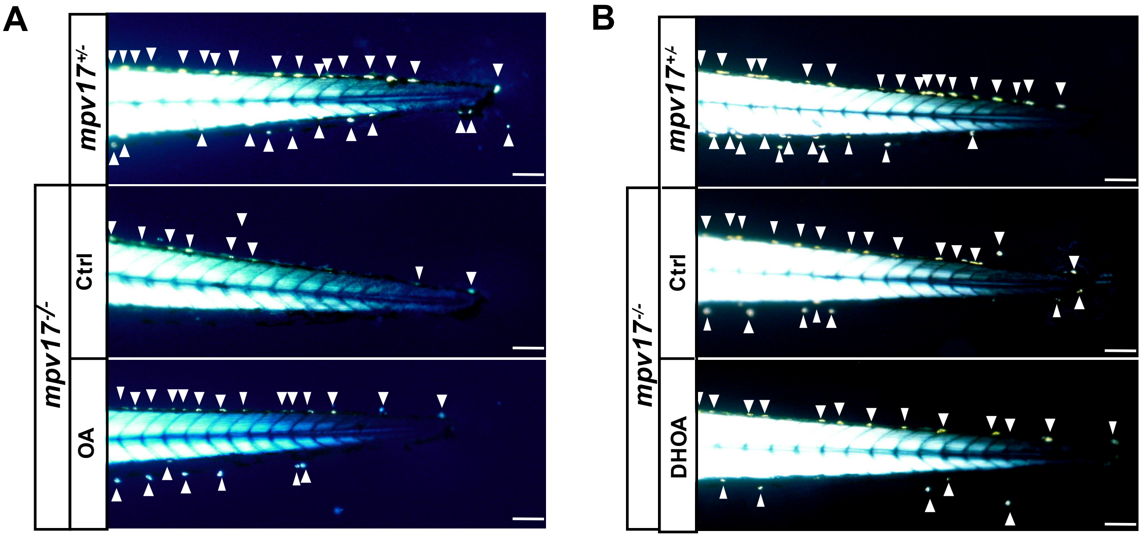

Figure S4. Administration of pyrimidine precursors to *mpv17* heterozygous and homozygous mutants and evaluation of iridophore phenotype at 3 dpf. Tail region birefringent image of 3 dpf larvae treated with 1 µM OA (A) and 100 µM DHOA (B). The arrows point to iridophores. Bars, 100 µm.

Disease Models & Mechanisms • Supplementary information
